# A Study of the Interaction of Bovine Hemoglobin with Synthetic Dyes Using Spectroscopic Techniques and Molecular Docking

**DOI:** 10.3389/fchem.2016.00050

**Published:** 2017-01-10

**Authors:** Kamaljeet, Saurabh Bansal, Uttara SenGupta

**Affiliations:** Department of Chemistry, Lovely Professional UniversityPhagwara, India

**Keywords:** fluorescence, molecular docking, UV-Visible spectroscopy, hemoglobin, azo-dyes, ligand binding

## Abstract

Synthetic dyes are a very efficient class of dyes that are ingested or come into contact with the skin from numerous sources (cosmetics, textiles, leather, paper, and drugs). An important component of their safety profile is the interactions that they form after they enter the body. Hemoglobin is a functionally important protein that can form multiple interactions with soluble compounds present in the blood, and hence forms an important aspect of the toxicological or safety profile of the dyes. Here we study the interaction between bovine hemoglobin and organic dyes using UV-Vis absorbance and fluorescence spectroscopy. Molecular modeling was used to visualize the binding site and partners of the dye molecules, within the hemoglobin molecule. We find that all four dyes studied form sufficiently strong interactions with hemoglobin to allow for the formation of potentially toxic interactions. Molecular modeling showed that all four dyes bind within the central cavity of the hemoglobin molecule. However, binding partners could not be identified as multiple binding conformations with very similar energies were possible for each dye.

## Introduction

Synthetic dyes are present in a very large number of products in daily use that are ingested (food, pharmaceuticals) or come into contact with our skin (cosmetics, textiles, leather, paper) and enter the body via the mouth, eyes, any cuts, or abrasions on the body. Inside the body, dye molecules enter the blood stream, where they have been shown to have the ability to enter cells (Kaji et al., 1991), including RBCs and interact with biomolecules present in the body, especially with proteins present in cells, tissue, and blood. The interaction of dyes with macromolecules present in the body forms a very important component of safety/toxicological profiles of these compounds. Biomolecular interactions are particularly important when dealing with OTC medicines, pharmaceuticals drugs, and their contra-indications. However, other common compounds found in items of daily use can also cause immunological and toxic reactions. Synthetic dyes are a very effective class of dyes. Of these, azo dyes form a major group of synthetic dyes and one of the most commonly used for their numerous hues and shades, fade resistance and strong attachment to fabrics (Yilmaz et al., 2007). The International Agency for Research on Cancer (IACR) has classified a number of azo dyes as carcinogenic due to the carcinogenicity of the aromatic amines formed as their degradation products in the body (Chung, 1983; Opinion of the Scientific Committee on Cosmetic Products and Non-Food Products Intended for Customers, 1997–2004; IACRs, 2016). Scientists have studied the interactions of a number azo dyes with proteins like hemoglobin and serum albumin. Changes in protein conformation as a result of protein–dye interaction can lead to change/loss of biological function of the protein, leading to toxicity (Chatterjee and Kumar, 2016). A number of studies show that azo dyes can show genotoxic, mutagenic, and developmental effects. Tissue culture experiments and mice models show significant developmental effects (Tsuda et al., 2001; An et al., 2007; Tanaka et al., 2008; Hashem et al., 2010).

**Figure 1:**
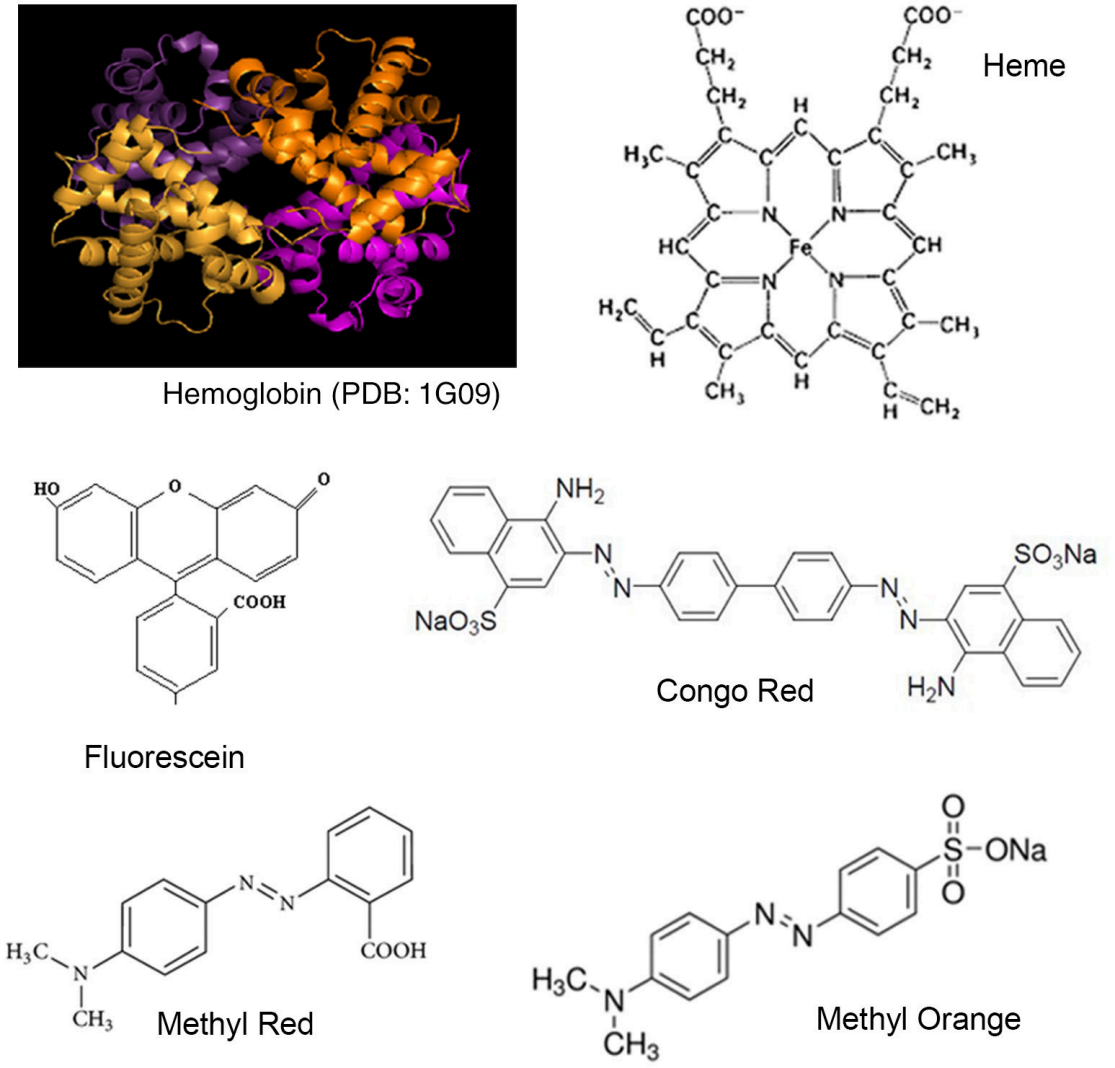
**Structures of Hemoglobin and synthetic dyes**.

Hemoglobin is a Fe (II)-protein found in the red-blood corpuscles of vertebrates, and is involved in the transport of oxygen and carbon dioxide between the lungs and tissues in the body. Forming a part of blood, hemoglobin can potentially interact with dyes as they are absorbed, and transported in the body. Hemoglobin is a well-known model system for protein studies and has been well-characterized using bio-physical methods. The quaternary structure of hemoglobin consists of a globular structure with four α-β sub-units (Figure 1A). Each sub-unit has α- or β-polypeptide chain attached to a heme component. Heme consists of an iron ion held in heterocyclic porphyrin ring (Figure 1B). Each α-chain consists of 141 amino acids, and each β-chain consists of 146 amino acids. The crystal structure of bovine hemoglobin at 2.1 A show few small structural and functional differences from the human hemoglobin structure (Mozzarelli et al., 1991; Safo and Abraham, 2001). On entering the body, drugs can interact with hemoglobin affecting their distribution and efficacy, and also forming the basis of potential toxicity (Messori et al., 2006). Hemoglobin has also been extensively used for studies in protein-ligand interactions with molecules for studies in toxicity and safety (Basu and Kumar, 2015; Fennell et al., 2015; Liu and Liu, 2015; Maltas and Ozmen, 2015).

In this report, we look at the interactions between hemoglobin and four organic dyes (fluorescein, congo red, methyl red, and methyl orange; Figure 1). Fluorescein is a very widely used dye and Congo red, Methyl red, and Methyl orange are azo dyes. Safety issues have been reported regarding all four dyes (Fineschi et al., 1999; Hitosugi et al., 2004; Sharma et al., 2005; Jalandoni-Buan et al., 2010; Wang et al., 2010; Wang and Zhang, 2012; Li et al., 2014,^1^). The strength of the binding interactions formed between Hemoglobin and each dye was determined from UV-Visible spectroscopy data using the Benesi-Hildenbrand equation (Benesi and Hildebrand, 1949; Nienhaus and Nienhaus, 2005). Complex formation was further investigated using Fluorescence spectroscopy (Alpert et al., 1980). The conformation of each dye molecule in the Hemoglobin-dye complex and Hemoglobin amino acid residues forming the binding site was investigated using molecular modeling (Autodock vina, Trott and Olson, 2010).

## Materials and methods

Hemoglobin in powder form was purchased from HIMEDIA laboratories (CAS No. 9008-02-0) and used without further purification. Congo red (Product No. 034002) and fluorescein (Product No. 028406) was obtained from CDH (Central Drug House). Methyl red (Catalog No. M41609) and methyl orange (Catalog No. M41309) was obtained from NICE-chemicals. All dyes were used without further purification. NaH_2_PO_4_.2H_2_O (CAS:13472-35-0, Loba Chemie) and NaH_2_PO_4_.2H_2_O (CAS:10028-24-7, Loba Chemie) were of analytical grade.

Hemoglobin was dissolved in 20 mM phosphate buffer (pH 7.2) and stored at 4°C. All dye stock solutions were prepared in distilled water and stored at 4°C. Binding reactions for data collection were prepared fresh in phosphate buffer (20 mM, pH 7.2).

### Apparatus and procedure

#### Spectroscopic studies

UV-vis absorption spectra were recorded on a Shimadzu UV-vis1800 (Japan). The wavelength range, 200–600 nm, was used for data collection. All fluorescence spectra were recorded on Perkin Elmer LS55 Fluorescence (U.S.A.) spectrophotometer. The excitation wavelength was set at 280 nm and emission data was collected between 300 and 500 nm. Excitation and emission slit widths were kept at 10.0 nm. Molecular docking was carried out using Autodock Vina (Maltas and Ozmen, 2015).

Two milliliters of individual reactions were set up for UV-Vis data collection. Fluorescence data was collected by successively titrating dye stock solution manually, and mixing the reaction by pipetting.

#### Molecular docking study

Methyl red, Congo red, Methyl orange, and fluorescein structures were obtained from the ZINC Database hosted at UCSF. The x-ray crystal structure for bovine Hemoglobin was taken from Protein Data Bank (PDB code 1G09). Molecular docking studies were carried out using the molecular docking software, Autodock Vina, from Scripps Research Institute, U.S.A. (Trott and Olson, 2010). Polar hydrogen atoms were added to the Hb crystal structure before starting the docking process, which required in addition only the assignment of the docking interface for the process.

## Results and discussion

### Analysis of UV-Visible spectroscopy

**Figure 2:**
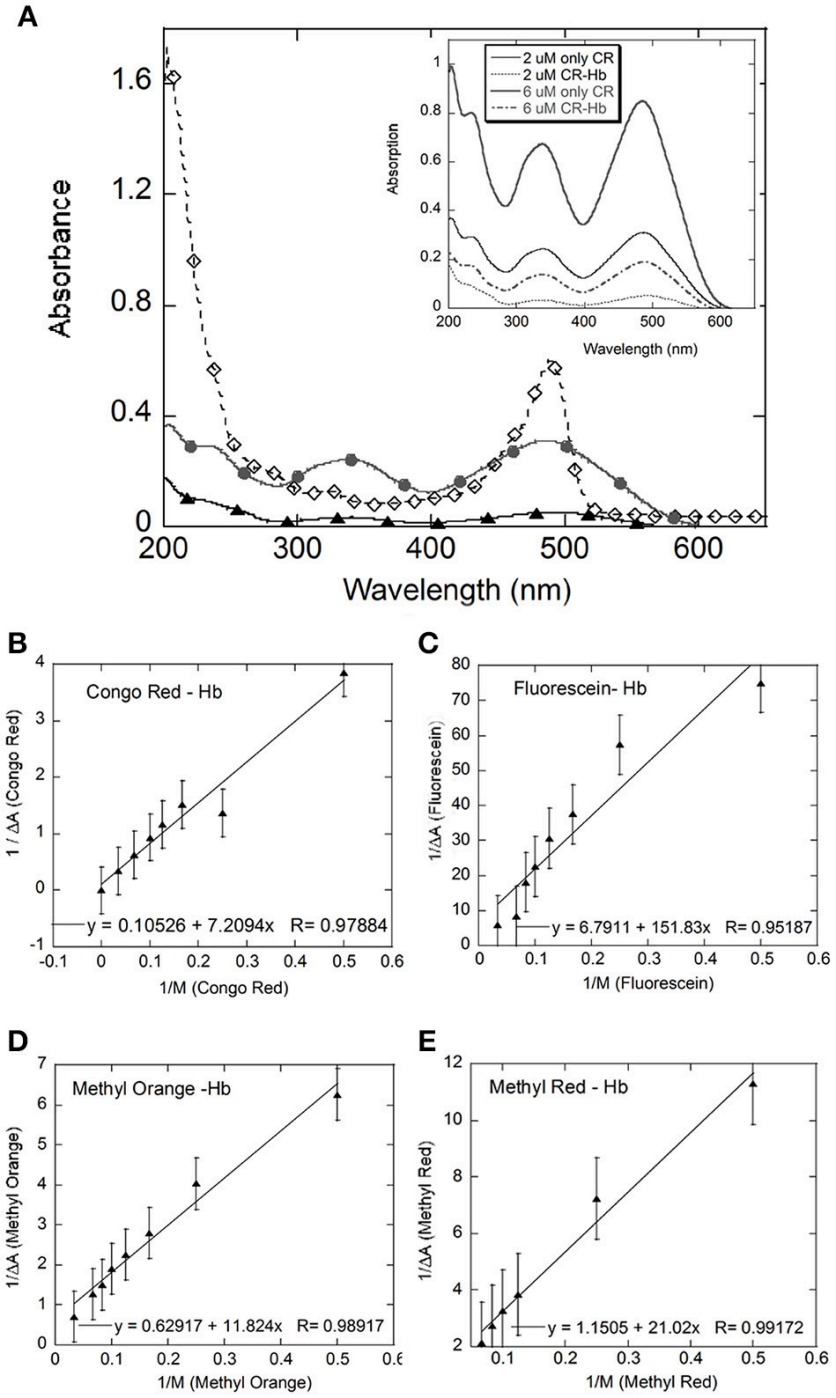
**UV-Visible spectral analysis**: **(A)** shows the UV-Visible spectra for Hb (black filled diamonds), Congo Red (CR, gray filled circles), and CR-Hb complex (filled triangles); Inset: Spectra of CR at 2 (gray line) and 6 μM (black line) and CR-Hb complex at 2 (----) and 6 μM (.....) CR. Benesi-Hildenbrand plots for all four dyes **(B–E)**. The concentration of Hb was kept constant at 5 μM and the dye concentration varied from 0 to 30 μM for Congo Red **(B)**, Fluorescein **(C)**, Methyl Orange **(D)**, and Methyl Red **(E)**.

Dyes which absorb in the visible range and proteins which absorb in the UV range can be studied using UV-Visible spectroscopy. The aromatic amino acids tryptophan, tyrosine and phenylalanine absorb in the 230–300 nm range and the peptide bond at ~230 nm. In the case of hemoglobin, the absorption spectra of the heme prosthetic group in hemoglobin is very sensitive to changes in polypeptide environment—structure, oxidation state, and ligand binding, rendering the technique highly suitable to follow Hb-ligand binding (Messori et al., 2006). Typically the hHb spectrum shows two major absorbance in the 199–700 nm range—a peak at ~200 nm due to the CO carbonyl groups in the amino acids and a Soret band seen at 405–450 nm due to the π–π transitions due to the heme moiety embedded in a hydrophobic pocket in the protein backbone (Messori et al., 2006; Chatterjee and Kumar, 2016). Our data (Figure 2) for bovine Hb showed a broad band in the 420–530 nm range, with its peak at 478 nm and a slight shoulder at ~450 nm. Bovine hemoglobin shows very few structural and functional differences from human Hb (Safo and Abraham, 2001). However, the high absorbance peak or the Soret band appears at 478 nm and the very weak α and β bands typically seen between 540 and 600 nm cannot be distinguished from the baseline (hematoporphyrin and fully oxygenated Hb: Soret band at 422–450 nm, β-band at 540 nm and α band at 576 nm) at the concentrations used (5–10 μM).

In this study, absorption for the dye (λ_max_) was followed using UV-Visible spectroscopy. The spectra for Congo Red (CR) is shown here as representative spectra (Figure 2A inset). Absorption data for CR from 200 to 800 nm, showed broad peaks with maxima at 343 and 486 nm. The broad peak around 485–490 nm could contain the azo-bond characteristic peaks at 493 and 488 nm (Wang and Zhang, 2012).

UV-visible spectra were taken of dye alone and of hemoglobin-dye complexes at increasing concentrations of dye. Changes in extinction coefficient and/or peak position of the dye on addition of the protein, or vice versa, are indicative of changes in the electronic environment of the molecule and hence complexation (Benesi and Hildebrand, 1949). The bands in the UV-Vis spectrum for the dye do not change position with the addition of Hb but show decreased absorption (Figure 2A inset), indicating interaction between the dye and Hb. Absorption of the complex showed ~86% deceased absorption relative to dye alone at 2 μM CR at 343 nm. This decrease in the absorption of the protein–dye complex was observed at all wavelengths between 200 and 600 nm, relative to the absorption spectrum of only dye. However, no significant change in peak positions in the spectrum of the complex relative to the spectrum of only dye was observed. In addition, the broad band with a peak at ~478 nm, seen in the Hb spectrum, shows decreased absorption with the addition of dye (data not shown), indicating a perturbation of the heme environment in the presence of dye (Franzen and Boxer, 1997; Dayer et al., 2010).

**Figure 3:**
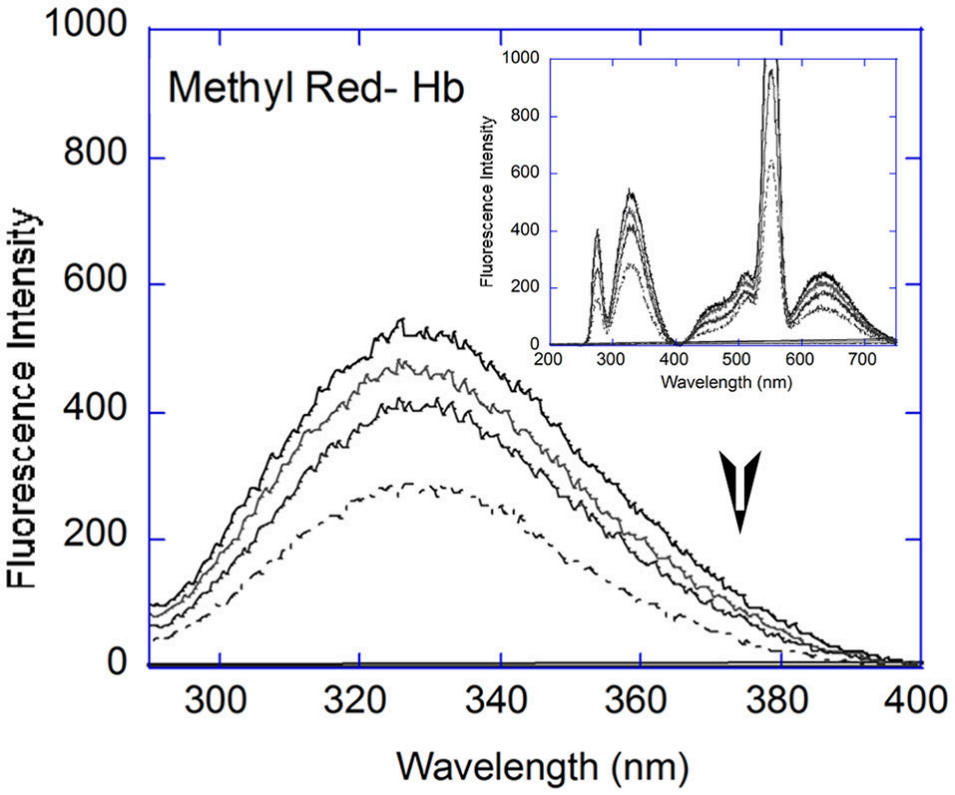
**Fluorescence Spectral analysis:** Fluorescence quenching with Hb (5 μM, black line) showed decreased emission with increasing concentrations of Methyl Red (2, 6, and 12 μM) in the 290–400 nm range. Inset: Full fluorescence spectrum (200–750 nm) at 2, 6, and 12 μM MR and 5 μM Hb (black line).

At each dye concentration, the absorption of only dye was subtracted from the absorption of the Hemoglobin–dye complex. The difference in absorption (Δ*A*) was then plotted against 1/[dye] (Figure 3) according to the Benesi-Hildenbrand equation (Fennell et al., 2015).

$$\begin{array}{l} \frac{1}{\Delta A}=\frac{1}{\left(\varepsilon_{b}-\varepsilon_{f}\right)L_{T}}+\frac{1}{\left(\varepsilon_{b}-\varepsilon_{f}\right)L_{T}K_{a}}\frac{1}{M} \end{array}$$

where ε is the extinction coefficient of bound and free states and *M* is protein concentration, *b* is bound ligand and *f* is free ligand and *T* is the total ligand concentration. Δ*A* is the change in absorbance at a given wavelength and *K*_*a*_ is the binding constant for the complex formation. Here, the value of *K*_*a*_ is given by the ratio of intercept to slope of the plot of 1/Δ*A* against 1/[dye] (Fennell et al., 2015). The values obtained for *K*_*a*_, for all four dyes were similar [Fluorescein = 0.44 (± 0.51) × 10^5^ M, Congo Red = 0.2 (± 1.17) × 10^5^ M, Methyl Red = 0.55 (± 0.22) × 10^5^ M, Methyl Orange = 0.53 (± 0.188) × 10^5^ M, Table 1].

**Table 1:** **Binding Parameters for Hb-dye complex**.

**Complex**	**Ka (Benesi-Hildenbrand)**	**Ksv (Stern-Volmer)**	**Kq**	**Ka (Modified Stern-Volmer)**	***n***
	**10^4^ M**	**10^4^M**	**10^11^ M L^−1^ s^−1^**	**10^4^ M**	
CR-Hb	0.15	5.4	5.4	0.77	0.73
Fl-Hb	0.44	6.2	6.2	5	0.18
MO-Hb	0.53	2.8	2.8	0.98	0.424
MR-Hb	0.55	9.3	9.3	0.49	1.15

The Benesi-Hildenbrand equation assumes that the decrease in absorption at dye (λ_max_) is entirely due to the formation of a 1:1 complex and hence is directly proportional to the concentration of the complex formed (Benesi and Hildebrand, 1949). A departure from linearity would indicate that the complex formed does not have the expected 1:1 protein–ligand stoichiometry or that less than expected ligand is available for complex formation. The linear fit for the data for all four dye molecules studied improves if data points at the concentration extremes are removed, removing the apparent non-linearity in the data. A non-linear model improves the fit to the data significantly only in the case of Fluorescein. The fit improves marginally in the case of Methyl Red and Methyl Orange, and not at all in the case of Congo Red (data not shown). The data for Fluorescein shows the greatest scatter and departure from a straight line. Fluorescein molecules are known to aggregate at higher concentrations which can lead to a lower than expected concentration of free Fluorescein being available for complex formation.

### Analysis of fluorescence spectroscopy

Parameters used to characterize an interaction between a protein and its ligand are its binding strength and the number of ligand binding sites on the protein. The intrinsic fluorescence of proteins, due to the amino acid residues tryptophan, tyrosine, and phenylalanine, change in response to conformational changes resulting from ligand/small molecule binding. Hemoglobin contains six Tryptophan amino acid residues, of which the β-37 Trp are the dominant fluorophores, sensitive to quaternary structural change (Alpert et al., 1980).

Quenching experiments were carried out with Hemoglobin and all four dyes. Quenching of Hemoglobin fluorescence was observed with increasing concentration of all four dyes. The fluorescent spectrum of hemoglobin with Methyl Red is shown as representative (Figure 3A). The Hemoglobin fluorescence emission peak between 290 and 400 nm showed quenching for all dyes studied and analyzed using Stern-Volmer and modified Stern-Volmer equations. The Stern-Volmer equation is given as:

$$\begin{array}{l} \frac{{\text{F}}_{\text{0}}}{\text{F}}=1+{\text{K}}_{\text{sv}}\left[\text{Q}\right] \end{array}$$

Where F is fluorescence intensity with quencher, F_0_ is fluorescence intensity without quencher, K_SV_ is the rate of quencher coefficient, and [Q] is the concentration of quencher (Trott and Olson, 2010). The modified Stern-Volmer equation is given:

$$\begin{array}{l} \text{lg}\frac{\left({\text{F}}_{\text{0}}-\text{F}\right)}{\text{F}}=\text{lgKa}+\text{nlg}\left[\text{Q}\right] \end{array}$$

Where F is fluorescence intensity with quencher, F_0_ is fluorescence intensity without quencher, K_a_ is equilibrium binding constant, n is the number of binding sites, and [Q] is concentration of quencher (Lakowicz, 1999). Fluorescence data was analyzed using both equations to derive the interaction parameters for binding between hemoglobin and each dye, i.e., the Stern-Volmer constant (K_sv_), the bimolecular quenching constant (K_q_), the binding constant (K_a_), and the number of binding sites (n, Figure 3). The results are shown in Table 1.

**Figure 4:**
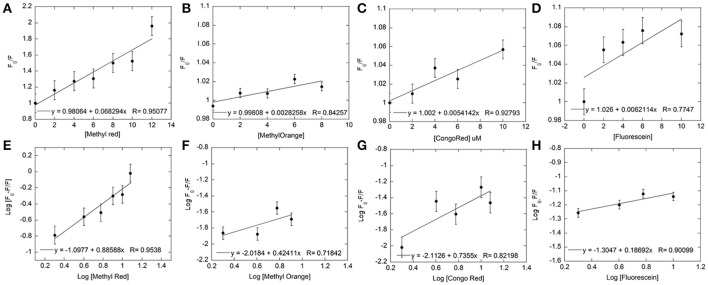
**Fluorescence quenching data for each dye was used to plot Stern-Volmer and Modified Stern-Volmer graphs**. The concentration of Hb was kept constant at 5 μM and the dye concentration varied from 0 to 12 μM for both Stern-Volmer **(A–D)** and Modified Stern-Volmer **(E–H)** graphs for Methyl Red **(A,E)**, Methyl Orange **(B,F)**, Congo Red **(C,G)**, and Fluorescein **(D,H)**. The linear graphs indicated the presence of only one type of quenching. Linear fits (shown within each graph) to the data were used to determine values for K_SV_, K_a_, and number of binding sites (n).

The Stern-Volmer plots are linear for all dyes under consideration here, indicating a single (static or dynamic) mode of fluorescence quenching. For each dye, the bimolecular collision constant K_q_ can be calculated from the value of K_sv_ and τ_0_ (average lifetime of Hb without quencher, 10^−10^s, Berezin and Achilefu, 2010). The maximum quenching constant K_q_ with biopolymers is 2 × 10^10^ M L^−1^ s^−1^. The calculated values of K_q_ for the four dyes are greater than the maximum value for collision quenching indicative of static quenching and complex formation (Ware, 1962). [Fluorescein = 0.006 (± 0.0029), Congo Red = 0.005 (± 0.0013), Methyl Red = 0.009 (± 0.001), Methyl Orange = 0.003 (± 0.0051), Table 1]. The scatter seen in the Stern-Volmer plot for Fluorescein (Figure 4D) may be in part due to the aggregation seen in Fluorescein, especially with increasing concentrations.

The association/binding constant for Hemoglobin-dye complexation (K_a_) and the number of binding sites (n) was determined from the intercept and slope, respectively, of the modified Stern-Volmer equation (given above), thus specifying the number of dye molecules bound to each hemoglobin molecule. Plots for both equations for all four dyes are shown in Figure 4. All four dyes showed similar association constants, which are within 2–6-fold of the association constants determined from UV-Visible experiments [Log Ka: Fluorescein = −1.3 (± 0.04), Congo Red = −2.113 (± 0.23), Methyl Red = −2.31 (± 0.22), Methyl Orange = −2.02 (± 0.72), Table 1]. The number of binding sites for each dye can be determined from the slope of the modified Stern-Volmer equation. The values shown in Table 1, indicate the expected single binding site for Congo Red (0.74 ± 0.3) and Methyl Red (1.16 ± 0.24). However, values for Fluorescein (0.19 ± 0.06) and Methyl Orange (0.424 ± 0.29) are unexpected and would indicate less than a full binding site for both dyes.

### Analysis of molecular docking

**Figure 5:**
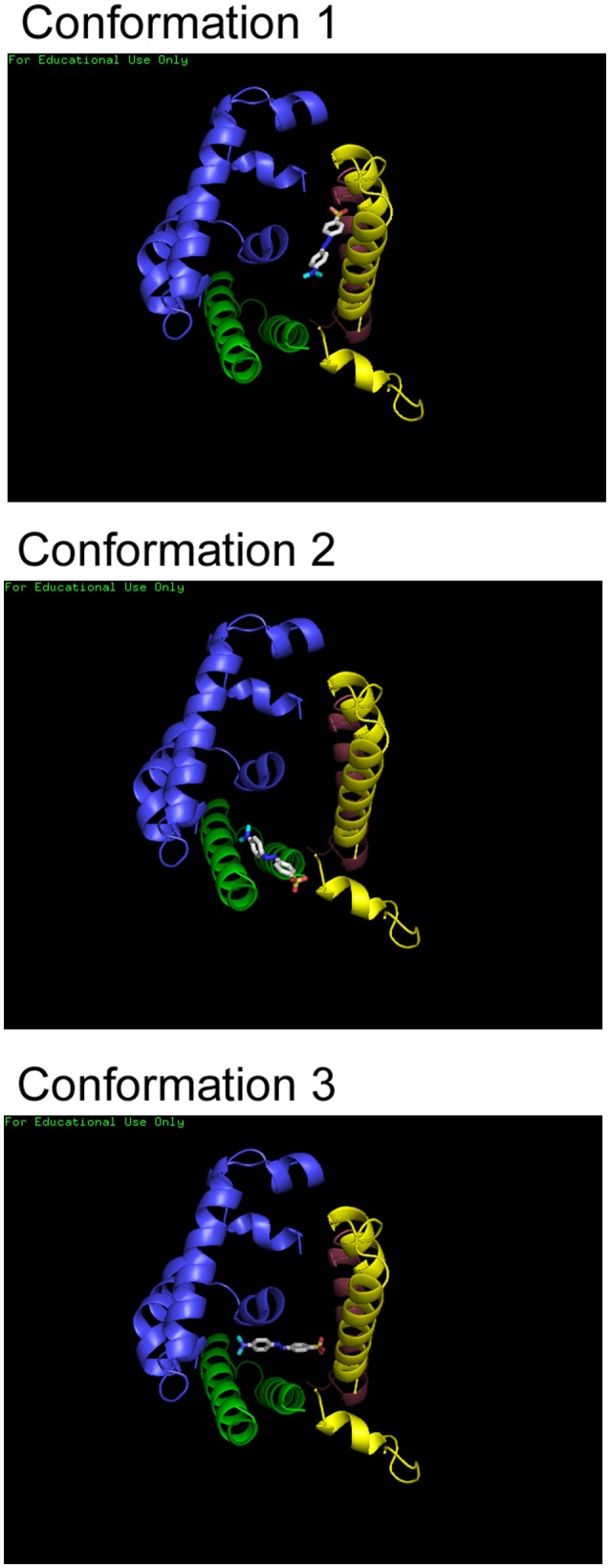
**Molecular docking of Hb and dyes**. Three of the lowest energy binding conformations that were predicted for Methyl Red are shown here. Structures were rendered using Pymol.

Molecular docking forms an important tool in drug design as it is allows estimation of the most stable binding conformation of small molecules to their target, hence allowing prediction of binding affinity, and activity of the molecule (Meng et al., 1992). The binding of Hemoglobin with each dye was simulated using the molecular docking software, Autodock Vina, from Scripps Research Institute, U.S.A. (Trott and Olson, 2010). The results of the docking program allows prediction of possible binding conformations of the ligand, binding site and binding partners for each dye with Hb. The results of the simulation showed multiple binding conformations (upto six different conformations) for each dye with very small differences in energy. The three lowest energy conformations for Methyl Red binding Hemoglobin have been shown as representative in Figure 5.

Interactions could be present between Methyl Red molecule and the same α- and β-chains of the protein. It is clear that all the dyes bind within the central cavity of the molecule. However, the exact binding partners or residues cannot be determined since a single binding conformation could not be identified. H-bonds and electrostatic interaction could be formed between the dye molecules and Hemoglobin residues falling within four angstroms be formed each conformation, stabilizing the hemoglobin-dye complex formed for all dyes.

## Conclusions

In this study, we investigate the binding of four synthetic dyes (Fluorescein, Congo Red, Methyl Red, and Methyl Orange) with bovine Hemoglobin, including two azo dyes (Methyl Red and Methyl Orange). We use Fluorescence and UV-Visible absorption spectroscopy to probe complex formation and molecular docking to simulate the binding interactions. The association constants determined from UV-Visible and Fluorescence spectroscopic studies were within 2–6-fold of each other, for each dye. The association constants were similarly high for all dyes, indicating that all dyes interacted and formed similarly strong complexes with Hemoglobin. Though, azo-dyes have a higher record of toxicity, the complexation of Congo Red and Fluorescein with hemoglobin also shows similar potential for toxicity. Fluorescence studies showed a single binding site for Congo Red and Methyl Red. However, the numbers for Fluorescein and Methyl Orange were much lower than one. This may be due to the multiple conformations via which the ligand (dye) interacts with the protein. Molecular docking studies done using Autodock Vina showed that all dyes bound HB within its central cavity, consistent with literature. However, multiple binding conformations were predicted for each dye, with very similar energies.

## Author contributions

Ka: M.Sc student who carried out Fluorescence and UV-Visible spectroscopy experiments, SB: B.Sc student who carried out UV-Visible spectroscopy experiments, US: Supervision and development of project, experiment and data collection, and writing of manuscript.

### Conflict of interest statement

The authors declare that the research was conducted in the absence of any commercial or financial relationships that could be construed as a potential conflict of interest.

## Footnotes

^1^*Material Safety Data Sheet Methyl orange from ScienceLab.com, Inc*. Available online at: http://www.sciencelab.com/msds.php?msdsId=9927361).
